# Current Data on Dietary Sodium, Arterial Structure and Function in Humans: A Systematic Review

**DOI:** 10.3390/nu12010005

**Published:** 2019-12-18

**Authors:** Christiana Tsirimiagkou, Eirini D. Basdeki, Antonios Argyris, Yannis Manios, Maria Yannakoulia, Athanase D. Protogerou, Kalliopi Karatzi

**Affiliations:** 1 Department of Medicine, Clinic & Laboratory of Pathophysiology, Cardiovascular Prevention & Research Unit, National and Kapodistrian University of Athens, 115 27 Athens, Greece; c.tsirimiagkou@med.uoa.gr (C.T.); eirinibasdeki@gmail.com (E.D.B.); and1dr@gmail.com (A.A.); 2 Department of Nutrition and Dietetics, School of Health Science and Education, Harokopio University of Athens, 176 76 Athens, Greece; manios@hua.gr (Y.M.); myianna@hua.gr (M.Y.); pkaratzi@hua.gr (K.K.)

**Keywords:** dietary sodium, arterial structure, arterial function, arteriosclerosis, arterial stiffness, arterial remodeling, arterial hypertrophy, atheromatosis, arterial plaques

## Abstract

Background: Subclinical arterial damage (SAD) (arteriosclerosis, arterial remodeling and atheromatosis) pre-exists decades before cardiovascular disease (CVD) onset. Worldwide, sodium (Na) intake is almost double international recommendations and has been linked with CVD and death, although in a J-shape manner. Studies regarding dietary Na and major types of SAD may provide pathophysiological insight into the association between Na and CVD. Objectives: Systematic review of data derived from observational and interventional studies in humans, investigating the association between dietary Na with (i) atheromatosis (arterial plaques); (ii) arteriosclerosis (various biomarkers of arterial stiffness); (iii) arterial remodeling (intima–media thickening and arterial lumen diameters). Data sources: Applying the PRISMA criteria, the PubMed and Scopus databases were used. Results: 36 studies were included: 27 examining arteriosclerosis, four arteriosclerosis and arterial remodeling, three arterial remodeling, and two arterial remodeling and atheromatosis. Conclusions: (i) Although several studies exist, the evidence does not clearly support a clinically meaningful and direct (independent from blood pressure) effect of Na on arterial wall stiffening; (ii) data regarding the association of dietary Na with arterial remodeling are limited, mostly suggesting a positive trend between dietary Na and arterial hypertrophy but still inconclusive; (iii) as regards to atheromatosis, data are scarce and the available studies present high heterogeneity. Further state-of-the-art interventional studies must address the remaining controversies.

## 1. Introduction

Cardiovascular disease (CVD) is responsible for 31 percent of all deaths worldwide (WHO 2018). The onset of CVD is preceded for decades by subclinical vascular functional and/or structural alterations, leading to transient or permanent subclinical arterial damage (SAD). Major types of SAD include atheromatosis (arterial atheromatic plaque formation), arteriosclerosis (arterial stiffening due to loss of the arterial wall’s elastic properties) and arterial remodeling (changes in arterial wall and lumen dimensions to maintain mechanical homeostasis). All the above modifications may occur simultaneously or separately.

In the last decade, a range of reliable, non-invasive vascular biomarkers have been used to detect SAD. Carotid ultrasonography is widely used to detect structural changes in the arterial wall (such as arterial plaques, indices of arterial remodeling, e.g., carotid intima–media thickness (cIMT) and arterial lumen diameters) [1,2,3]. On the other hand, arterial stiffening is classically measured by applanation tonometry to obtain carotid–femoral pulse wave velocity (cfPWV), the gold standard for clinical practice, although other methods have been used [3]. The study of these vascular biomarkers provides the opportunity to not only optimize CVD risk classification but also to elucidate the pathogenesis and pathophysiology of CVD in the early clinical steps.

Globally, sodium (Na) intake is almost double (mean intake: 3.95 g/day) [4] the recommended levels by the World Health Organization (less than 2 g/day) [5]. High Na intake has been strongly correlated with CVD [6,7,8]. Moreover, there is strong evidence from large-scale studies of a blood pressure (BP)-lowering effect (by 3.39 mmHg for systolic BP and 1.54 mmHg for diastolic BP)—and consequently CVD-risk lowering effect—after a reduction in Na intake to less than 2 g/day compared to an intake higher than 2 g/day [8]. However, very low levels of Na intake (approximately below 1.5 g/day) have also been linked to increasing CVD risk, suggesting a J-shaped trend [9,10,11,12,13,14]. Although the effect of salt on BP is variable due to salt sensitivity subtypes, consideration of this heterogeneity has been neglected in previous meta-analyses [9,10]. Several observational and/or interventional studies have tested the association of Na intake with types of SAD, but there are still many contradictory results and questions to be addressed [15,16]. Most data derive from studies investigating the relationship between Na and arterial stiffness or hypertrophy, suggesting that higher levels of Na intake are positively associated with these types of SAD [17,18,19,20,21], although this has not been seen consistently [16,17,18]. Studies regarding dietary Na and atheromatosis are scarce and are limited by major methodological issues [19,20].

In an attempt to better understand the potential associations that link dietary Na intake and SAD, the aim of this systematic review is to evaluate—for the first time—data from observational and interventional studies in humans, investigating associations between dietary Na intake and SAD as well as the effect of Na intake on SAD-related changes. All types of SAD were considered, namely (i) atheromatosis (arterial plaques); (ii) arteriosclerosis (arterial stiffening); (iii) arterial remodeling (intima–media thickening and arterial lumen diameters).

## 2. Materials and Methods 

This study was prepared and reported in accordance with Preferred Reporting Items for Systematic Reviews and Meta-Analyses (PRISMA) statement [21] (Appendix A).

### 2.1. Search Strategy

A systematic search of potentially relevant studies was performed through July 2019 by two separate reviewers on the PUBMED and SCOPUS databases. Search terms applied were: ((“sodium intake” or “na intake” or “na+ intake” or “sodium excretion” or “na excretion” or “na+ excretion” or “dietary sodium” or “dietary na” or “dietary na+” or “urinary sodium” or “urinary na” or “urinary na+”)) and (“arterial function” or “vascular function” or “arterial structure” or “vascular structure” or plaque or atheroma or “atheromatic plaque” or “atherosclerotic plaque” or atheromatosis or atherosclerosis or arteriosclerosis or “arterial remodeling” or “carotid plaque” or “femoral plaque” or “arterial stiffness” or “arterial stiffening” or “pulse wave velocity” or pwv or “intimal medial thickness” or “intima media thickness” or IMT or “wall to lumen ratio”). Studies were limited to the English language and human studies. Reference lists of included articles were also examined for additional relevant articles.

### 2.2. Inclusion and Exclusion Criteria

The following inclusion criteria were applied: relevant epidemiological studies or clinical trials, English language, human studies, males and/or females of any age regardless of diseases (chronic or acute), clearly described outcome defined as: association between Na intake and/or excretion with atheromatosis (presence of plaques), arteriosclerosis (any accepted biomarker of arterial stiffening at any arterial segment) or arterial remodeling (arterial hypertrophy (IMT) or artery lumen diameters). The following exclusion criteria were applied: epidemiological studies with a sample <100 subjects, animal studies, reviews, systematic reviews, meta-analyses, comments/letters, studies using the assessment of Na intake and/or excretion of biomarkers other than Na (e.g., the ratio Na/K).

### 2.3. Selection of Studies and Data Extraction

Two reviewers screened the available titles, abstracts and keywords of all the available articles. Discrepancies were resolved after discussion. After agreement, full text screening was carried out. Qualitative and quantitative data from all included articles were extracted by both reviewers. The extracted data included specific details for study design, population characteristics, Na estimation method and outcomes related to Na and vascular damage. All units of Na are presented as mg (converted from mmol to mg, if necessary). Predefined variables (shown in Table 1, Table 2, Table 3, Table 4 and Table 5) were extracted. 

## 3. Results

### 3.1. Number of Studies Screened and Selected

**Figure 1:**
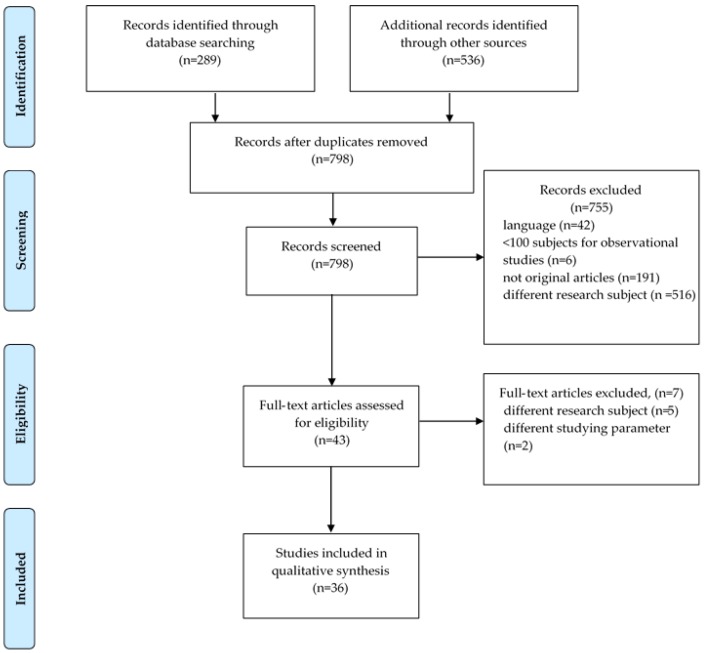
PRISMA flow diagram.

Eight hundred and twenty-five (825) citations were identified through a systematic search—of which, 782 were excluded on the basis of title/abstract. The most common exclusion criteria were: language (42), duplicates—same cohort (27), different research subject (516), not original articles (191), sample size <100 subjects for observational studies only (6). Forty-three (43) articles were then assessed for eligibility and five were excluded due to irrelevant research subject and two due to different studying parameter (Na/K ratio). As a result, the number of articles that met the inclusion criteria and were included in this study were 36 (Figure 1).

From a total of 36 studies included, 18 were observational and 18 were interventional studies. Detailed descriptive data for both observational and interventional studies are provided in Table 1, Table 2, Table 3, Table 4 and Table 5.

### 3.2. Description of Studies

Population description and exclusion criteria are reported in Appendix B.

#### 3.2.1. Studies Investigating Arteriosclerosis (Arterial Stiffness)

Thirty-one (31) studies examining arteriosclerosis were identified—of which, 14 were observational [15,16,17,18,19,20,27,28,29,30,31,32,33,34] and 17 were interventional [22,23,35,36,37,38,39,40,41,42,43,44,45,46,47,48,49].

##### Observational Studies

From the observational studies investigating arteriosclerosis, 11 out of 14 found a positive association between arterial stiffness biomarkers and dietary Na [15,17,18,19,20,28,29,30,32,33,34], one out of 14 found a J–shaped association [23], one out of 14 found an inverse association [16] and one out of 14 found no association [27] (Table 1). From the above 11 studies showing a positive association, nine of them measured vascular parameters at one time point [17,18,19,20,28,29,30,33,34] and the remaining two studies evaluated arterial stiffness at two different time points [15,28].

Heterogeneity in the assessment of arterial stiffness existed in the above 11 studies [15,17,18,19,20,28,29,30,32,33,34] due to: (a) various arterial stiffness biomarkers using different methodologies (four applanation tonometry [17,28,29,30], six oscillometry [15,18,19,32,33,34] and one b-mode ultrasonography [33]) at different arterial segments using various arterial stiffness biomarkers (five cfPWV [17,28,29,30,32], one aortic PWV other than cfPWV [29], four baPWV [15,30,31,32] and one common carotid artery elasticity (Young’s elastic modulus, stiffness index, arterial compliance) [33]) (Table 1); (b) various Na assessment methods (seven studies used 24h urine collection [17,18,19,28,29,30,34], two spot urine collections [28,29] and two a combination of dietary methods [15,33]); (c) different populations (five hypertensives [24,25,31,32,33], one normotensive [26], one chronic kidney disease patient [28], three mixed populations [15,22,29] and one healthy subjects [30]) (Table 1).

Moreover, one out of the 11 studies showed that high Na excretion (mean: 2898 mg/day, range 2035.5–3588) is associated with cfPWV only when high Na excretion was combined with high renin–angiotensin–aldosterone system (RAAS) activity but not in the other groups (i.e., those with high Na and low RAAS, low Na and low RAAS, as well as low Na and high RAAS) [24].

Only seven out of these 11 studies adjusted the results for BP level [17,18,19,20,29,30,34] and only three of them persistently showed a positive association between arterial stiffness and Na after the adjustment [22,31,32].

In the one study that showed an inverse association between arterial stiffness and Na, the result persisted after adjustment for BP level [16].

Finally, salt sensitivity assessment was not conducted in any of the above 14 observational studies.

##### Interventional Studies

From the 17 interventional studies investigating the association between arteriosclerosis [22,23,35,36,37,38,39,40,41,42,43,44,45,46,47,48,49], seven of them showed statistically significant changes in arterial stiffness biomarkers after Na intake intervention [35,37,38,44,45,46,49]. On the contrary, 10 out of the 17 interventional studies found no changes in arterial stiffness biomarkers with various levels of Na intake during the intervention [22,23,36,39,40,41,42,43,47,48] (Table 2).

In detail, three out of seven that found significant changes showed that increases in dietary Na were associated with an increase in arterial stiffness biomarkers [37,43,45] and four out of the seven showed that a reduction in dietary Na intake was associated with a decrease in arterial stiffness biomarkers [34,36,44] or even an increase in arterial elasticity biomarkers [48] (Table 2). Three of these seven studies found statistically significant changes only in specific intervention groups [36,43,45] (one study found that reduced Na excretion was associated with a decrease in cfPWV only in blacks, but not in whites and Asians [36]; one study found that high Na intake was associated with increased hfPWV only in salt-sensitive but not in salt-resistant participants [45]; one study found that a high-salt diet was associated with increased cfPWV only in middle-aged participants and not in young participants [43]).

In those seven studies finding statistically significant changes in PWV after high or low-Na diets [35,37,38,44,45,46,49], heterogeneity existed, regarding: (a) different methodologies used for arterial stiffness assessment (three b-mode ultrasonography [34,45,48], one oscillometry [44] and three tonometry [36,37,43]) and different arterial stiffness biomarkers assessed (four cfPWV [34,36,37,43], one aortic PWV other than cfPWV [44], one heart-femoral (hfPWV) [45] and one arterial compliance [48]); (b) various methodologies used for Na assessment (four combination of dietary and urinary methods [34,37,44,48], two 24h urine collection [36,43] and one not available data [45]); (c) different duration of intervention period and (d) different populations (four in hypertensives or subjects with high normal BP [34,36,37,48], two in normotensives [43,44] and one in mixed populations (hypertensives and normotensives) [45]) (Table 2).

Of note, out of the seven studies that found statistically significant associations between Na and arterial stiffness biomarkers [35,37,38,44,45,46,49] only three studies adjusted the results for BP level [34,43,45]. One out of the three studies found that the statistically significant association between high-Na diet (6900 mg/day) and cfPWV in middle-aged adults was lost after correcting for the mean BP level [43]. Both other two studies found that their findings were independent from mean BP level [34,45].

In the 10 studies that found no statistically significant changes in arterial stiffness biomarkers after different levels of Na intake [22,23,36,39,40,41,42,43,47,48], heterogeneity existed, regarding: (a) different methodologies used for arterial stiffness assessment (seven tonometry [22,23,39,40,41,42,43], one oscillometry [35], one plethysmography [46] and one diastolic blood pressure time decay method [47]) using similar arterial stiffness biomarkers assessed (eight cfPWV [22,23,36,39,40,41,42,43], one baPWV [46] and one arterial compliance [47]); (b) various methodologies used for Na assessment (eight 24h urine collection [22,39,40,41,42,43,47,48] and two combination of dietary and urinary methods [18,35]); (c) different duration of intervention period and (d) different population samples (six in hypertensives [17,35,38,40,41,46] (one in overweight or obese hypertensives [35], one in hypertensives with chronic kidney disease patients [38], three in hypertensives [17,41,46], one in prehypertensives [40]), two in normotensives [18,47], one in overweight or obese subjects [39] and one in women with preeclampsia or healthy pregnancy in the past [42])) (Table 2).

Finally, only two out of the 17 conducted salt sensitivity assessment [45,46]. One out of the two studies revealed that the result was not statistically significant in the salt-resistant group, but only in the salt-sensitive group [45]. On the contrary, in the other study no significant differences between Na interventions and PWV were revealed for both salt-sensitive and salt-resistant participants, but salt-sensitive participants had higher baPWV at each time point of the intervention (baseline, low-Na diet, high-Na diet) [46].

#### 3.2.2. Studies Investigating Arterial Remodeling

Nine studies examining arterial remodeling were identified [15,16,19,20,23,33,49,50,51]—of which, eight were observational [15,16,19,20,23,33,49,50] (Table 3) and one was interventional [51] (Table 4).

##### Observational Studies

Out of the eight observational studies, six found positive [15,19,20,33,49,50], one inverse [16] and one J-shaped associations [23] between cIMT and Na intake or excretion (Table 3). Out of the eight observational studies, seven measured the outcome at one time point (cross-sectional) [16,19,20,23,33,49,50] and one study measured the outcome at two time points and examined the association between the change of cIMT and Na intake (prospective) as well [15] (Table 3). In the prospective study, although the cIMT was positively associated with Na intake, the change of cIMT during follow up was negatively associated with Na intake [15] (Table 3). Four out of the six studies that found positive associations between cIMT and Na adjusted their results for BP level [19,33,49,50]: in two of them, the result was no more statistically significant after adjustment for BP [33,49], in one of the studies, the result was marginally not significant after BP adjustment [50] and in the remaining one, the result was independent from BP [19]. The remaining two studies did not adjust their results for BP level [15,20]. Finally, one out of the six studies that found a positive association implied a statistically significant correlation only with IMT at the carotid bifurcation but not at the common carotid artery [19].

Heterogeneity in the assessment of arterial remodeling existed in the above six studies due to: (a) different Na assessment methods (four dietary (one [19]) or a combination of dietary (three [15,20,33]) methods, two 24h urine collection [49,50]) and (b) different studied populations (chronic diseases, age, comorbidities). All studies assessed cIMT as arterial remodeling biomarker via b-mode ultrasonography excluding from the measurement arterial segments with atheromatic plaques (Table 3).

The only study showing an inverse association was the only one conducted in purely normotensives as well as the only one using spot urine specimens for Na assessment [16]. Adjustment for BP was performed in this study and the result was independent from BP level [16]. The only study which showed a J-shaped association did not adjust the results for BP level [23].

Salt sensitivity assessment was not conducted in any of the eight studies.

##### Interventional Studies

The only interventional study that investigated the association between Na intake and arterial remodeling (Table 4) used brachial and carotid artery diameter as end point [51]. The brachial artery lumen increased after 8 weeks of a low-Na diet (mean ± SD: 1955 ± 220.8 mg/day) but no changes in the common carotid diameter were revealed. The findings were adjusted for BP levels [51]. Salt sensitivity assessment was not conducted [51] (Table 4).

#### 3.2.3. Studies Investigating Atheromatosis

Only two observational studies examining atheromatosis were identified, showing conflicting results [19,20] (Table 5). One study showed that higher Na intake (2050–2330 mg/day vs. 780–900 mg/day) is positively associated with the prevalence off carotid plaques [20], while the other study did not find a statistically significant association [19]. The two studies assessed Na via different ways (dietary and urinary) and used different populations (elderly females [20] as well as a general population [19]). Both studies examined carotid plaques via B-mode ultrasonography. One out of the two studies adjusted their results for BP levels and the result was independent from BP [19]. No study assessed salt sensitivity.

## 4. Discussion

In the present study, we performed a systematic review of the literature to investigate the relationship between dietary Na intake with arterial function and structure using gold-standard non-invasive vascular biomarkers to measure arteriosclerosis, arterial remodeling and atheromatosis. The results of this systematic review indicate that: (i) although several studies have investigated the association of dietary Na with arterial stiffness, the evidence does not clearly support a clinically meaningful, direct and independent from BP effect of Na on the arterial wall to increase arterial stiffness; (ii) data regarding the association between dietary Na and arterial remodeling are limited, mostly suggesting a positive trend between dietary Na and arterial hypertrophy, but still inconclusive; (iii) data regarding the association between dietary Na and atheromatosis are scarce and the available studies present high heterogeneity.

### 4.1. Na and Arteriosclerosis

Although 31 human studies have investigated the association between dietary Na and arteriosclerosis, the current data are inconclusive regarding a potential direct effect of Na on the arterial wall properties that accelerate the arterial stiffening process. Indeed, the majority of the studies (observational 11/14 and interventional 7/17) do imply the presence of a harmful effect of high Na intake [15,17,18,19,20,28,29,30,32,33,34,38,44,46] or even benefits of low Na intake on arterial stiffening parameters [34,36,44,48] (18 out of 31, 11 observational and seven interventional), in various populations [22,26,29,34,44], involving several different segments of the arterial tree [22,29,30,33,45,48], independently of the applied methodology, technology used [22,29,33,34]. However, most of these positive studies do not take into consideration the well-known effect of Na on BP increase [15,24,28,29,36,37,44]. Overall only 1/3 of the studies included in our analysis, and only 10 out of the 17 positive studies adjusted their findings for BP levels [17,18,19,20,29,30,34,35,44,46]. Even more interestingly, in more than half of them (six out of 10), the association between Na and indices of arteriosclerosis was lost after correcting for BP [25,26,30,33,34,43]. Moreover, although salt sensitivity is a major factor modulating the effect of Na on BP (and therefore to arterial stiffness), only two [45,46] out of the 31 studies evaluated this parameter and showed conflicting results. Indeed, there is evidence suggesting that a high-salt diet would increase BP in 17% of the subjects (salt sensitives), reduce BP in 11% (inverse salt sensitive) and not significantly affect BP in the remaining salt-resistant subjects [52]. Finally, just one study [23] showed a J-shaped association between Na and arteriosclerosis, mirroring the recent epidemiological data on the J-shaped association between Na and mortality. 

A recent meta-analysis of randomized controlled trials, conducted by D’ Elia and colleagues [53], being the first and the only one available on this topic so far, included 14 cohorts (all of them included in our work) and showed a statistically significant decrease by 2.84% in cfPWV after an average reduction of approximately 2 g (89.3 mmol) per day in Na intake independently from BP. In this meta-analysis, the authors excluded all the studies measuring other than the cfPWV, whereas we extended our systematic review to include all valid non-invasive indices of arterial stiffness including other segments of the arterial bed (such as the carotid artery and the lower limbs). Although our study is not applying a synthesis of quantitative data (as a meta-analysis), but uses only the qualitative characteristics of the selected studies, it is important to consider that the result of D’ Elia et al. suggest poor, if any, clinical effects of Na on arterial stiffness. A reduction of PWV by 2.84% may not offer additional benefit in overall vascular health.

Taken all together, these data suggest that arterial stiffness can be reduced with a dietary intervention aiming at the reduction of dietary Na intake, but: (a) this reduction is modest (e.g., aortic stiffness of 10 m/s considered the high CVD risk cut-off level will be reduced to 9.8 m/s after a major reduction of Na by 2 g/day) with debatable clinical effect and (b) it is not established whether this lowering effect is mediated only by BP reduction or mediated by a direct effect on the arterial wall [54,55].

Moreover, major questions seek suitable answers, since poor data regarding the role of salt sensitivity, the RAAS, age and race exist. The hypothesis that hyperactive RAAS leads to BP elevation and consequently arterial stiffening, as a result of BP rising in salt-sensitive subjects, cannot yet be rejected. In a single study, Kotliar et al. indicate a significant positive association between Na and PWV only in the group of participants who had high RAAS activity. However, the group with high Na and low RAAS activity did not show a significant association with PWV [24]. One of the studies suggested that only middle-aged and not young participants presented increased PWV after a high-salt diet [43]. However, in the study by Avolio et al., all of the age groups (children, young adults and middle-aged adults) decreased their PWV after reducing Na intake [44]. Finally, despite the fact that race has been shown to play a significant role in BP levels and salt sensitivity, indirectly affecting arterial stiffening, just one study addressed this issue and showed significant increases in PWV after Na supplementation only in black participants.

### 4.2. Na and Arterial Remodeling

Nine studies—all of them using B-mode ultrasonography—investigating the association between dietary Na and arterial remodeling were identified (eight observational [15,16,19,20,23,33,49,50] and one interventional [51]). The majority of them (six out of the nine) implied a detrimental effect after high Na intake [15,19,20,33,49,50] or even a beneficial effect after low Na intake [51] on arterial remodeling parameters (cIMT or artery diameters) independently of different methods used for Na assessment and various population groups (different diseases and comorbidities, age groups, etc.). In most cases, higher dietary Na intake was associated with higher cIMT in plaque-free arterial segments, mostly at the common carotid, suggesting arterial hypertrophy, but also carotid bulb [19] and brachial artery [51]. 

However, only three out of the nine studies included large population samples (>1500 participants) [15,16,19] and their results were conflicting, since one of them found an inverse and BP-independent association between Na and cIMT but was the only one conducted in purely normotensives and assessed Na through spot urine specimens as well [16]. Probably, the best available study so far, the only interventional study published by Benetos et al., showed that independently from BP, increased Na intake only induced arterial remodeling in a muscular artery (brachial artery) but not in an elastic one (carotid artery), suggesting a diverging effect of Na in different arterial beds [51]. 

Most importantly, once more, the effect of potential confounding BP on arterial remodeling was not taken into consideration in 1/3 of the studies (three out of nine) [15,20,23]. Further, in two other studies [33,49], the end point was actually mediated by BP increase. In conclusion, data on the association between dietary Na and arterial remodeling are limited, mostly suggesting a positive trend between dietary Na and arterial hypertrophy, but this is still inconclusive and conflicting. No study assessed salt sensitivity.

To our knowledge, the association between Na and arterial remodeling has not previously been subject to meta-analysis, and despite positive trends observed in the majority of studies, there is insufficient data to conclusively establish the relationship.

### 4.3. Na and Atheromatosis

According to our systematic research, there are extremely limited data on the association between dietary Na and atheromatosis. Only two studies examined this association [19,20]. Mazza et al., in a very small study [20], found that high dietary Na is associated with the increased prevalence of carotid plaques, whereas Dai et al., in a substantially larger study [19], suggested a non-significant association between dietary Na intake and carotid plaques. However, these studies presented heterogeneity in population samples (elderly females [20] and general population [19]), sample size (108 [20] and 3290 [19] participants) and Na assessment method (24h dietary recall and 7 day food record [20] and FFQ [19]). Moreover, the available studies regarding dietary Na and atheromatosis have not investigated the association between very low and very high levels of Na, and that might explain why a J-shaped trend has not been observed. Furthermore, beyond carotid arteries, plaque formation in other arterial segments that might offer an additive value in CVD prevention—such as the femoral arteries—has not been assessed in any of the available studies. In conclusion, there is not enough evidence to support a positive, negative or J-shaped association between dietary Na and arterial plaques and more studies investigating the association between larger ranges of Na intake/excretion and arterial plaques are needed.

### 4.4. Strengths and Limitations

Major strengths of our study are: (i) the novel concept of investigating the effect of dietary Na on SAD, including all the major pathogenetic mechanisms (arteriosclerosis, arterial remodeling & atheromatosis); (ii) the systematic nature of this review in order to compare and dispose all the available international literature on this specific topic; (iii) the design of our study, including clinical trials and evidence from observational studies in order to investigate the short- and long-term effects of different levels of Na intake on SAD. A limitation of our study is the absence of a quantitative analysis of the extracted data (meta-analysis), which could lead to a clearer view of the topic. 

## 5. Conclusions

In conclusion, there is not yet enough evidence to support a direct and causal association between Na and each of the major types of SAD, even in the most widely studied case of arteriosclerosis (arterial stiffening). The available data derive mostly from small, heterogeneous, not well-designed studies. Especially in the case of arterial remodeling and atheromatosis, both common and clinically relevant types of structural arterial damage have scarcely been investigated in relation to Na intake or excretion. One of the dominant issues is the heterogeneity of the studies in Na assessment method. Precise quantification of Na intake is difficult and despite the fact that only the 24h urine collection is regarded as a gold standard, based on the knowledge that approximately 90% of Na intake is excreted through urine, other dietary or spot urinary methods are commonly used in studies. Several disadvantages of the above mentioned studies have been described, such as underreporting, equations suitable only for specific population groups, different recipes, etc., leading to inaccurate measurements. Finally, many studies included in our analysis do not address the cardinal effect of Na on BP and almost all of them neglect the role of salt-sensitivity. Future studies using novel diagnostic tests for individuals’ salt sensitivity assessment are needed to clarify the role of dietary Na to SAD [56]. More well-designed interventional studies are needed in order to resolve all the remaining controversies.

## Figures and Tables

**Table 1:** Descriptive characteristics of observational studies regarding arteriosclerosis.

ARTERIOSCLEROSIS													
**cfPWV**													
**1. Observational Cross-Sectional Studies**													
**Author (Year)**	**Country**	**Study Design**	**Population Description**	**FU (Years)**	**Sex**	**Race**	**N**	**Age (Years, Mean ± SD)**	**Na Estimation Method**	**Na Intake/Excretion (mg/d)**	**Vascular Assessment**	**Results**	
Polónia, J. (2006) [22]	Portugal	c-sect	essential HT, recent stroke or healthy university students	-	M/F	Mixed	426	50 ± 22	24hU	Total: 4646 ± 1472	tonometry	+ **	
García-Ortiz, L. (2012) [23]	Spain	c-sect	primary care patients aged 30–80	-	M/F	N/AV	351	54.8 ± 11.7	FFQ	Total: 3180 ± 1250 Q1:1800 ± 390 Q2: 2650 ± 200 Q3: 3440 ± 270	tonometry	J-shaped curve	
Kotliar, C. (2014) [24]	Argentina	c-sect	essential HT, aged 30 to 70	-	M/F	N/AV	300	48.7 ± 14.6	24hU	(a) low Na-low RAAS: 913.1 (747.5–1035)	tonometry	≠	
										(b) low Na-high RAAS: 690 (602.6–740.6)		≠	
										(c) High-Na-low RAAS: 2610.5 (1745.7–3604.1)		≠	
										(d) high-Na-high RAAS: 2898 (2035.5–3588)		+ *	
Polonia, J. (2016) [25]	Portugal	retrosp	HT adults	7.2 (0.5–11.1)	M/F	White	608	54.1 ± 14.3	24hU	4793.2 ± 1821.6	tonometry	+ *	
Strauss, M. (2018) [26]	South Africa	prosp	NT adults	-	M/F	Mixed	693	24.8 ± 3.01	24hU	2967 (984.4–7613)	tonometry	total	+ **
												black	+ *
												white	≠
Triantafyllou, A. (2018) [27]	Greece	c-sect	untreated HT—healthy individuals	-	M/F	White	197	43.7 ± 12.1	24hU	True HT: 3348.8 (2251.7–4595.4) Intermediated HT phenotypes: 3128 (1902.1–4312.5) NT: 2732.4 (1630.7–4312.5)	tonometry	≠	
**2. Observational Studies with Follow Up (>1 Time Points)**													
**Author (Year)**	**Country**	**Study Design**	**Population Description**	**FU (Years)**	**Sex**	**Race**	**N**	**Age (Years, Mean ± SD)**	**Na Estimation Method**	**Na Intake/Excretion (mg/d)**	**Vascular Assessment**	**Results**	
Nerbass, F.B. (2015) [28]	UK	prosp	adults in CKD stage 3	1	M/F	N/AV	1607	72.6 ± 9.0	Spot urine collections and Nerbass equation to estimate 24h Na excretion	baseline: 2599 ± 782 follow-up: 2576 ± 782	oscillometry	**cfPWV** (at both time points)	
												Na <2300 mg	+ **
												Na >2300 mg	≠
												**ΔcfPWV**	
												Unchanged Na	≠
												Decreased Na	≠
												Increased Na	+ *
**Aortic PWV Other than cfPWV**													
Siriopol, D. (2018) [29]	Romania	prosp	HT and NT Romanian adults	-	M/F	White	1599	47.3 ± 17.1	Morning spot urine sample + Kawasaki equation	4816.2 ± 1550.2	oscillometry	NT	+ **
												HT	+ **
**baPWV**													
**1. Observational Cross-Sectional Studies**													
Sonoda, H. (2012) [30]	Japan	c-sect	healthy subjects	-	M/F	Asian	911	61.3 ± 8.5	24hU	720 ± 200 (mg /day/10 kg)	oscillometry	+ **	
Lee, S.K. (2015) [16]	Korea	c-sect	non-HT subjects, with no use of anti-HT drugs	-	M/F	Asian	1586	tertile 1: 52.1 ± 5.5 tertile 2: 53.0 ± 6.0 tertile 3: 52.6 ± 5.5	Second morning void and Tanaka’s equation to convert to 24hU	3588 ± 782	plethysmography	− **	
Sun, N. (2015) [31]	China	c-sect	newly diagnosed HT, untreated HT or patients with a 1 to 5 year history of HT who had stopped taking anti-HT drugs for 1 month	-	M/F	N/AV	341	Group A: 59.3 + 13.4 Group B: 56.1 + 15.5 Group C: 57.6 + 14.2	24hU	Total: 3507.5 ± 1577.8 Group A: 1807.8 ± 411.7 Group B: 3374.1 ± 618.7 Group C: 5858.1 ± 961.4	oscillometry	+ *	
**Author (year)**	**Country**	**Study Design**	**Population Description**	**FU (years)**	**Sex**	**Race**	**N**	**Ag (Years, Mean ± SD)**	**Na Estimation Method**	**Na Intake/Excretion (mg/d)**	**Vascular Assessment**	**Results**	
Han, W. (2017) [32]	China	c-sect	HT adults	-	M/F	N/AV	431	Group A: 54.5 ± 12.9 Group B: 52.9 ± 12.6 Group C: 50.9 ± 11.4	24hU	Total: 3831.8 ± 1.610, Group A: 1768.7 ± 464.6 Group B: 3371.8 ± 650.9 Group C: 5947.8 ± 1069.5	oscillometry	+ *	
**2. Observational Studies with Follow Up (>1 Time Points)**													
Jung, S. (2019) [15]	South Korea	prosp	adults aged >40	5.3±1.0	M/F	Mixed	2145	59.9 ± 9.1	FFQ and 3 day diet record	2538 ± 1416	oscillometry	**baPWV**	
												Na baseline	+ **
												Na average of three visits	+ **
												**ΔbaPWV**	
												Na baseline	+ **
												Na average of three visits	+ **
**Common Carotid Arterial Elasticity (Young’s Elastic Modulus, Stiffness Index, and Arterial Compliance)**													
Ferreira-Sae, M.C. (2011) [33]	Brazil	c-sect	HT adults	-	M/F	N/AV	134	58 ± 1	1. FFQ 2. 24h recall 3. discretionary Na intake ^1^	Na intake/d: 5520 ± 290 FFQ: 1450 ± 180 24h recall: 940 ± 70 Discretionary Na: 3130 ± 190	B-mode US	Young’s elastic modulus	+ **
												stiffness index	≠
												arterial compliance	≠

The mmol of Na intake/excretion values were converted to mg. If available, results presented come from adjusted models. Abbreviations: Na: sodium; 24hU: 24h urine collection; cfPWV: carotid–femoral pulse wave velocity; baPWV: brachial–ankle pulse wave velocity; HT: hypertensives; NT: normotensives; anti-HT: antihypertensive; c-sect: cross-sectional; prosp: prospective; retrosp: retrospective; FFQ: food frequency questionnaire; FU: follow up; M/F: males and females; N/AV: not available; RAAS: renin–angiotensin–aldosterone system; CKD: chronic kidney disease; US: ultrasonography; +: positive association; -: negative association; ≠: no statistically significant association; *: *p* < 0.05; **: *p* < 0.01. ^1^ number of 1 kg packages of salt consumed/month/person.

**Table 2:** Descriptive characteristics of interventional studies regarding arteriosclerosis.

ARTERIOSCLEROSIS															
**cfPWV**															
**Author**	**Country**	**Study Design**	**Population Description**	**Sex**	**Race**	**N**	**Age (Years, Mean ± SD)**	**Intervention Duration (Weeks)**	**Type of Diet**	**Na Estimation Method**	**Na Intake/Excretion (mg/d)**		**Vascular Assessment**	**Results**	
														**Intervention Groups**	**cfPWV Change (m/s)**
Seals, D.R. (2001) [34]	USA	RCT	postmenopausal women, ≥50 years, high normal SBP or Stage 1 HTN	F	Mixed	17	65 ± 10	13	LS < 2400 mg	24hU & food records	Urinary Na excretion Preint_restr: 2852 ± 1058 Postintn_restr:1978 ± 736 Dietary Na intake Preint_restr: 2685 ± 559 Postint_restr:1421 ± 512		US	LS vs. baseline	−0.24 *
Dickinson, K.M. (2009) [35]	Australia	cross-over RCT	OW/OB, mild HT adults	M/F	N/AV	29	52.7 ± 6.0	4 (2 weeks × two diets)	Usual Na diet: 3450 mg Na/d vs. LS diet: 1150 mg Na/d	Three-day weighed food records & 24hU	Urinary method		oscillometry	LS vs. Usual Na	≠
											Baseline	3553.5 ± 1568.6			
											Usual Na	3594.9 ± 1304.1			
											LS	1474.3 ± 949.9			
He, F.J. (2009) [36]	UK	cross-over dbRCT	HT adults	M/F	Mixed	169	All: 50 ± 11 Blacks: 50 ± 9 Whites: 52 ± 12 Asians: 47 ± 10	12	9 Na tablets (×230 mg)/d & 9 placebo tablets/d. (remained on LS diet: 2000 mg Na/d)	24hU	Total	3013 ± 1150	tonometry	From Na to placebo	
														Total	−0.40 **
											Blacks	3036 ± 1058		Blacks	−0.50 **
											Whites	2921 ± 1173		Whites	≠
											Asians	3174 ± 1311		Asians	≠
Pimenta, E. (2009) [17]	USA	cross-over RCT	resistant HT adults on a stable anti-HT drug	M/F	Mixed	12	55.5 ± 9.4	2 (1 week × two diets)	LS diet: 1495 mg Na/d vs. HS diet: suppl. >5750 mg Na/d	24hU	Baseline: 4478.1 ± 1577.8 LS diet: 1060.3 ± 616.4 vs. HS diet: 5800.6 ± 1485.8		tonometry	From HS to LS ≠	
Todd, A.S. (2010) [37]	New Zealand	cross-over sbRCT	PHT or HT, NOB adults or on anti-HT drugs	M/F	Mixed	33	51.8 ± 7.6	12	(500 mL tomato juice + LS diet/day) (A) 0 + 1380 mg (B) 2070 + 1380 mg (C) 3220 + 1380 mg	Morning spot urine samples & dietary recalls	Na intake		tonometry	B vs. A	+ 0.39 **
											Usual diet	2607 ± 1289		C vs. A	+ 0.35 **
											A	1254 ± 397		B vs. C	≠
											B	1357 ± 486			
											C	1306 ± 335			
Todd, A.S. (2012) [18]	New Zealand/Australia	cross-over sbRCT	NT, NOB adults	M/F	N/AV	23	43.7 (24–61)	12	(500 mL tomato juice + LS diet/day) (A) 0 + 1380 mg (B) 2070 + 1380 mg (C) 3220 + 1380 mg	Morning spot urine samples & dietary recalls	Pre-baseline:	2410.4	tonometry	B vs. A	≠
											After intervention (Na intake + Na tomato juice)			C vs. A	≠
											A	0 + 1232.8			
											B	2070 + 1207.5		B vs. C	≠
											C	3220 + 1140.8			
McMahon, E.J. (2013) [38]	Australia	cross-over dbRCT	HT adults, with stage 3 or 4 CKD	M/F	N/AV	20	68.5 ± 11	4 (2 weeks × two diets)	HS diet: 4140–4600 mg Na/d vs. LS diet: 1380–1840 mg Na/d	24hU	LS: 1725 (1334–2576) vs. HS: 3864 (3358–5037)		tonometry	LS vs. HS	≠
Dickinson, K.M. (2014) [39]	Australia	cross-over sbRCT	OW or OB subjects	M/F	N/AV	25	N/AV	6	LS diet: 2400 mg/d vs. Usual Na diet: 3600 mg/d	24hU	baseline: 2761 ± 1031 Usual Na diet: 1729 ± 627 LS diet: 1799 ± 497		tonometry	LS vs. US	≠
Gijsbers, L. (2015) [40]	the Netherlands	cross-over RCT	untreated (P)HT, aged 40–80	M/F	White	36	65.8 (47–80)	4	Na suppl: 3000 mg/d vs. placebo	24hU	Baseline: 3535.1 Na suppl.: 4666.7 ± 1260.4 vs. Placebo: 2417.3 ± 913.1		tonometry	Na suppl. vs. placebo	≠
Suckling, F.J. (2016) [41]	United Kingdom	Cross-over dbRCT	untreated HT adults	M/F	Mixed	46	58 ± 1	12 (6 weeks × two diets)	9 Na tablets (×230 mg)/d vs. 9 placebo tablets/d.	24hU	Na diet: 3797.3 ± 207 vs. Placebo: 2681.8 ± 218.5		tonometry	Na diet vs. placebo	≠
van der Graaf, A.M. (2016) [42]	the Netherlands	cross-over RCT	women with history of preeclampsia or history of healthy former pregnancy	F	N/AV	36	36 ± 5	2	LS diet: 1150 mg/d vs. HS diet: 4600 mg/d	24hU	NT pregnancy history group: LS: 897 ± 322 HS: 5083 ± 1472 Preeclamptic pregnancy history group: LS: 1035 ± 529 HS: 5934 ± 1978		tonometry	LS vs. HS (in either group)	≠
Muth, B.J. (2017) [43]	USA	cross-over RCT	healthy, NT adults	M/F	N/AV	85	Young: 27 ± 1 Middle-aged: 52 ± 1	2	LS diet: 460 mg/d vs. HS diet:6900 mg/d	24hU	* LS diet (young & middle aged): 690 HS diet (young & middle-aged): 5405 * Approximately from diagram		tonometry	middle aged	+0.60 **
														young	≠
**Aortic PWV (Other than cfPWV)**															
**Author**	**Country**	**Study Design**	**Population Description**	**Sex**	**Race**	**N**	**Age(Years, Mean ± SD)**	**Intervention Duration**	**Type of Diet**	**Na Estimation Method**	**Na Intake/Excretion (mg/d)**		**Vascular Assessment**	**Results**	
														**Intervention groups**	**PWV Change (%)**
Avolio AP. (1986) [44]	Australia	RCT	healthy NT adults and children	M/F	N/AV	114	Group 1: Control: 10.8 ± 1.9 LS: 10.4 ± 2.5 Group 2: Control: 39.4 ± 1.7 LS: 39.8 ± 1.6 Group 3: Control: 52.2 ± 3.5 LS: 54.5 ± 4.2	24.8±2.5 months (8 months to 5 years)	N/AV	24hU & diet questionnaire	Na excretion: Control group: N/AV Group 1: 1564 Group 2: 943 Group 3: 506		oscillometry	Group 1 leg	−11.2 *
														Group 2 aortic arm leg	−21.8 ** −10.7 * −13.3 *
														Group 3 aortic leg	−22.7 * −22.3 *
**hfPWV**															
Rhee MY. (2016) [45]	Korea	RCT	NT and HT adults	M/F	N/AV	101	46.0 ± 16.6	2	LS DASH diet: 2320 mg Na/d vs. HS DASH diet: 7000 mg Na/d	N/AV	LS diet: 2320 vs. HS diet: 7000		US	HS vs. LS	
														SS	+4% *
														SR	≠
														HT	≠
														NT	≠
**baPWV**															
Wang Y. (2015) [46]	China	dietary intervention study	mild HT adults	M/F	N/AV	49	49.0 ± 7.9	3 (1 week × three diets)	LS diet: 1179.9 mg/d & HS diet: 7079.4 mg/d	24hU	3999.7 ± 1543.3		plethysmography	LS vs. HS	≠
														SS vs. SR	
														Baseline	+2.3 *
														After LS	+1.5 *
														After HS	+2.0 *
**Arterial Elasticity (Arterial Compliance)**															
**Author**	**Country**	**Study Design**	**Population Description**	**Sex**	**Race**	**N**	**Age (Years, Mean ± SD)**	**Intervention Duration**	**Type of Diet**	**Na Estimation Method**	**Na Intake/Excretion (mg/d)**		**Vascular Assessment**	**Results**	
														**Intervention Groups**	**Vascular Change (mm/mmHg)**
Creager MA. (1991) [47]	USA	cross-over RCT	NT men	M	N/AV	17	30 ± 2 years	10 days	LS diet: 230 mg Na/d vs. HS diet: 4600 mg Na/d	24hU	LS: 253 ± 46 vs. HS: 4117 ± 207		diastolic blood pressure time decay method	LS vs. HS	≠
Gates PE. (2004) [48]	USA	cross-over dbRCT	stage 1 HT adults, older than 50	M/F	White	12	men: 63 ± 1 women: 64 ± 4	8 weeks	LS diet: 1196 ± 92 & Normal Na diet: 1311 ± 23	3 day dietary records & 24hU	Na excretion Baseline: 3105 LS: 1380 Normal Na: 3450		B-mode US	LS	+0.04 *
														Normal	≠

The mmol of Na intake/excretion values were converted to mg. If available, results presented come from adjusted models. Abbreviations: Na: sodium; 24hU: 24h urine collection; cfPWV: carotid–femoral pulse wave velocity; baPWV: brachial–ankle pulse wave velocity; hfPWV: heart-femoral pulse wave velocity; HT: hypertensives; NT: normotensives; PHT: pre-hypertensives; HTN: hypertension; SBP: systolic blood pressure; anti-HT: antihypertensive; OW: overweight; OB: obese; NOB: non-obese; suppl: supplementation; HS: high sodium; LS: low sodium; SS: salt sensitive; SR: salt resistant; RCT: randomized controlled trial; sbRCT: single-blind RCT; dbRCT: double-blind RCT; M/F: males & females; F: females; M: males; N/AV: not available; CKD: chronic kidney disease; US: ultrasonography; ≠: no statistically significant association; *: *p* < 0.05; **: *p* < 0.01.

**Table 3:** Descriptive characteristics of observational studies regarding arterial remodeling.

Arterial Remodeling													
cIMT													
**1. Observational Cross-Sectional Studies**													
**Author (Year)**	**Country**	**Study Design**	**Population Description**	**FU (Years)**	**Sex**	**Race**	**N**	**Age (Years, Mean ± SD)**	**Na Estimation Method**	**Na Intake/Excretion (mg/d)**	**Vascular Assessment**	**Results**	
Ferreira-Sae, M.C. (2011) [33]	Brazil	c-sect	HT adults	-	M/F	N/AV	134	58 ± 1	1. FFQ 2. 24h recall 3. discretionary Na intake ^1^	Na intake/d: 5520 ± 290 FFQ: 1450 ± 180 24h recall: 940 ± 70 Discretionary Na: 3130 ± 190	B-mode US	+ *	
Njoroge, J.N. (2011) [49]	USA	c-sect	OW or OB, physically inactive adults	-	M/F	Mixed	258	Total: 38.5 ± 5.8 Q1: 39.3 ± 5.6 Q2: 38.7 ± 5.2 Q3: 37.6 ± 6.2 Q4: 38.2 ± 6	24hU	Total:1104–9545 Q1: 1104–3289 Q2: 3312–4117 Q3: 4140–5152 Q4: 5175–9545	B-mode US	+ *	
García-Ortiz, L. (2012) [23]	Spain	c-sect	primary care patients aged 30–80	-	M/F	N/AV	351	Total: 54.8 ± 11.7 Q1: 57.6 ± 12.1 Q2: 55.9 ± 11.3 Q3: 54.7 ± 10.5	FFQ	Total: 3180 ± 1250 Q1:1800 ± 390 Q2: 2650 ± 200 Q3: 3440 ± 270	B-mode US	J-shaped	
Lee SK. (2015) [16]	Korea	c-sect	non-HT subjects, with no use of anti-HT drugs	-	M/F	Asian	1586	tertile 1: 52.1 ± 5.5 tertile 2: 53.0 ± 6.0 tertile 3: 52.6 ± 5.5	second morning void & Tanaka’s equation	3588 ± 782	B-mode US	− **	
Ustundag, S. (2015) [50]	Turkey	c-sect	ambulatory adult patients, in stage 2–4 CKD	-	M/F	N/AV	193	Na excretion <1955 mg/day: 47.7 ± 10.6 ≥1955 mg/day: 49.7 ± 11.0 Mean IMT <0.750 mm: 45.1 ± 12.2 ≥0.750 mm: 52.3 ± 8.3	24hU	<1955 mg/day: 3220 ± 69 ≥1955 mg/day: 3220 ± 69 Mean IMT < 0.750 mm: 3220 ± 46 Mean IMT ≥ 0.750 mm: 3220 ± 69	B-mode US	+ **	
Dai, X.W. (2016) [19]	China	c-sect	Asian adults, via subject referral and community advertisement	-	M/F	Asian	3290	M: 62.1 ± 6.7 F: 59.4 ± 5.5	FFQ	Dietary Na intake: Q1: 833 ± 394 Q2: 864 ± 507 Q3: 825 ± 41 Q4: 828 ± 395	B-mode US	common cIMT	≠
												carotid bifurcation IMT	+ *
Mazza, E. (2018) [20]	Italy	c-sect	adults aged ≥65, not suffering from any debilitating diseases	-	F	White	108	70 ± 4	24h dietary recall + 7 day food record	1476 ± 618	B-mode US	+ *	
**2. Observational Studies with Follow up (>1 Time Points)**													
Jung, S. (2019) [15]	South Korea	prosp	adults aged >40	5.4 ± 1.0	M/F	Mixed	2494	60.2 ± 9.0	FFQ + 3 day diet record	2644 ± 1573	B-mode US	**cIMT**	
												Na baseline	≠
												Na average of three visits	+ **
												**ΔcIMT**	
												Na baseline	≠
												Na average of three visits	− *

The mmol of Na intake/excretion values were converted to mg. If available, results presented come from adjusted models. Abbreviations: Na: sodium; 24hU: 24h urine collection; cIMT: carotid Intima Media Thickness; HT: hypertensives; anti-HT: antihypertensive; OW: overweight; OB: obese; c-sect: cross-sectional; prosp: prospective; FFQ: food frequency questionnaire; FU: follow up; M/F: males & females; F: females; N/AV: not available; CKD: chronic kidney disease; US: ultrasonography; +: positive association; -: negative association; ≠: no statistically significant association; *: *p* < 0.05; **: *p* < 0.01. ^1^ number of 1 kg packages of salt consumed/month/person.

**Table 4:** Descriptive characteristics of interventional studies regarding arterial remodeling.

Arterial Remodeling														
Right Branchial Artery and Common Carotid Artery Diameter														
Author	Country	Study Design	Population Description	Sex	Race	N	Age (Years, Mean ± SD)	Intervention Duration (Weeks)	Type of Diet	Na Estimation Method	Na Intake/Excretion (mg/d)	Vascular Assessment	Results	
Benetos, A. (1992) [51]	France	cross-over dbRCT	actively working, mild to moderate HT adults	M/F	N/AV	20	41.5 ± 2.4	8	Group 1 and Group 2: Normal Na diet (NS diet, Na capsules): 1400 mg and low-Na diet (LS diet), lactose capsules	24hU	Baseline: 3979 ± 299 LS diet: 1955 ± 220.8 NS diet: 3749 ± 305.9	B-mode US	LS diet vs. NS diet	
													Brachial artery diameter	+0.67m **
													common carotid diameter	≠

The mmol of Na intake/excretion values were converted to mg. If available, results presented come from adjusted models. Abbreviations: sodium; 24hU: 24h urine collection; HT: hypertensives; LS: low sodium; dbRCT: double-blind RCT; M/F: males & females; N/AV: not available; US: ultrasonography; ≠: no statistically significant association; **: *p* < 0.01.

**Table 5:** Descriptive characteristics of observational studies regarding atheromatosis.

Atheromatosis												
Carotid Plaques												
Author (Year)	Country	Study Design	Population Description	FU (Years)	Sex	Race	N	Age (Years, Mean ± SD)	Na Estimation Method	Na Intake/Excretion (mg/d)	Vascular Assessment	Results
Dai, X.W. (2016) [19]	China	c-sect	Asian adults, via subject referral and community advertisement	-	M/F	Asian	3290	M: 62.1 ± 6.7 F: 59.4 ± 5.5	FFQ	Dietary Na intake: Q1: 833 ± 394 Q2: 864 ± 507 Q3: 825 ± 41 Q4: 828 ± 395	B-mode US	≠
Mazza, E. (2018) [20]	Italy	c-sect	Adults aged ≥65, not suffering from any debilitating diseases	-	F	White	108	70 ± 4	24h dietary recall + 7 day food record	1476 ± 618 Tertile I: 780–900 Tertile II: 1330–1430 Tertile III: 2050–2330	B-mode US	Tertile III vs. Tertile I + *

The mmol of Na intake/excretion values were converted to mg. If available, results presented come from adjusted models. Abbreviations: Na: sodium; c-sect: cross-sectional; FFQ: food frequency questionnaire; FU: follow up; M/F: males & females; F: females; US: ultrasonography; +: positive association; ≠: no statistically significant association; *: *p* < 0.05.

## Appendix A. PRISMA Checklist

**TITLE**			
Title	1	Identify the report as a systematic review, meta-analysis, or both.	1
**ABSTRACT**			
Structured summary	2	Provide a structured summary including, as applicable: background; objectives; data sources; study eligibility criteria, participants, and interventions; study appraisal and synthesis methods; results; limitations; conclusions and implications of key findings; systematic review registration number.	1
**INTRODUCTION**			
Rationale	3	Describe the rationale for the review in the context of what is already known.	1,2
Objectives	4	Provide an explicit statement of questions being addressed with reference to participants, interventions, comparisons, outcomes, and study design (PICOS).	1,2
**METHODS**			
Protocol and registration	5	Indicate if a review protocol exists, if and where it can be accessed (e.g., web address), and, if available, provide registration information including registration number.	Not applicable
Eligibility criteria	6	Specify study characteristics (e.g., PICOS, length of follow-up) and report characteristics (e.g., years considered, language, publication status) used as criteria for eligibility, giving rationale.	2
Information sources	7	Describe all information sources (e.g., databases with dates of coverage, contact with study authors to identify additional studies) in the search and date last searched.	2
Search	8	Present full electronic search strategy for at least one database, including any limits used, such that it could be repeated.	2,3
Study selection	9	State the process for selecting studies (i.e., screening, eligibility, included in systematic review, and, if applicable, included in the meta-analysis).	3
Data collection process	10	Describe method of data extraction from reports (e.g., piloted forms, independently, in duplicate) and any processes for obtaining and confirming data from investigators.	2
Data items	11	List and define all variables for which data were sought (e.g., PICOS, funding sources) and any assumptions and simplifications made.	2
Risk of bias in individual studies	12	Describe methods used for assessing risk of bias of individual studies (including specification of whether this was done at the study or outcome level), and how this information is to be used in any data synthesis.	Not applicable
Summary measures	13	State the principal summary measures (e.g., risk ratio, difference in means).	-
Synthesis of results	14	Describe the methods of handling data and combining results of studies, if done, including measures of consistency (e.g., I^2^) for each meta-analysis.	Not applicable
Risk of bias across studies	15	Specify any assessment of risk of bias that may affect the cumulative evidence (e.g., publication bias, selective reporting within studies).	Not applicable
Additional analyses	16	Describe methods of additional analyses (e.g., sensitivity or subgroup analyses, meta-regression), if done, indicating which were pre-specified.	Not applicable
**RESULTS**			
Study selection	17	Give numbers of studies screened, assessed for eligibility, and included in the review, with reasons for exclusions at each stage, ideally with a flow diagram.	3
Study characteristics	18	For each study, present characteristics for which data were extracted (e.g., study size, PICOS, follow-up period) and provide the citations.	4–15
Risk of bias within studies	19	Present data on risk of bias of each study and, if available, any outcome level assessment (see item 12).	Not applicable
Results of individual studies	20	For all outcomes considered (benefits or harms), present, for each study: (a) simple summary data for each intervention group (b) effect estimates and confidence intervals, ideally with a forest plot.	Not applicable
Synthesis of results	21	Present results of each meta-analysis done, including confidence intervals and measures of consistency.	Not applicable
Risk of bias across studies	22	Present results of any assessment of risk of bias across studies (see Item 15).	Not applicable
Additional analysis	23	Give results of additional analyses, if done (e.g., sensitivity or subgroup analyses, meta-regression [see Item 16]).	Not applicable
**DISCUSSION**			
Summary of evidence	24	Summarize the main findings including the strength of evidence for each main outcome; consider their relevance to key groups (e.g., healthcare providers, users, and policy makers).	15,16
Limitations	25	Discuss limitations at study and outcome level (e.g., risk of bias), and at review-level (e.g., incomplete retrieval of identified research, reporting bias).	18,19
Conclusions	26	Provide a general interpretation of the results in the context of other evidence, and implications for future research.	18,19
**FUNDING**			
Funding	27	Describe sources of funding for the systematic review and other support (e.g., supply of data); role of funders for the systematic review.	19

## Appendix B. Population Description and Exclusion Criteria of Selected Studies

**Arteriosclerosis—Observational Studies**		
**cfPWV**		
**1. Observational Cross-Sectional Studies**		
**Author (year)**	**Population Description**	**Exclusion Criteria**
Polónia, J. (2006)	essential hypertensives, recent stroke or healthy university students	urine sample not meeting the required quality criteria
García-Ortiz, L. (2012)	primary care patients aged 30–80	cardiovascular and/or cerebrovascular disease
Kotliar, C. (2014)	essential hypertensives, aged 30 to 70	abnormal renal function; volume or electrolyte alterations; diabetes mellitus; history of renal disease, ischemic heart disease, stroke; loss of data; counter indication for the drug washout; use of corticoids or nonsteroidal anti-inflammatory drugs during the study
Polonia, J. (2016)	hypertensive adults	secondary hypertension, previous cardiovascular events history; estimated glomerular filtration rate >50 mL/min/1.73
Strauss, M. (2018)	normotensive adults	previously diagnosed chronic illness (self-reported); use of anti-hypertensive drugs or other chronic diseases; diabetes mellitus; HIV infected; microalbuminuria>30 mg/mL; pregnancy or lactation
Triantafyllou, A. (2018)	newly diagnosed & never-treated hypertensives—healthy individuals admitted for regular check-up	previously treated with anti-hypertensive drugs; use of any kind of drugs; other known health problems; secondary causes of hypertension; other comorbidities (e.g., diabetes mellitus, CVD)
**2. Observational Studies with Follow up (>1 Time Points)**		
Nerbass, F.B. (2015)	adults in CKD stage 3	solid organ transplant or terminally illness
**Aortic PWV Other Than cfPWV**		
Siriopol, D. (2018)	hypertensive & normotensive Romanian adults	use of diuretic treatment; CKD; missing values for the variables of interest
**baPWV**		
**1. Observational Cross-Sectional Studies**		
Sonoda, H. (2012)	healthy subjects	heart failure; valvular heart disease; atrial fibrillation; peripheral artery disease
Lee, S.K. (2015)	non-hypertensive subjects, with no use of anti-hypertensive drugs	age >70 years; preexisting CVD including significant valvular heart diseases and arrhythmia; chronic renal disease or a serum creatinine level >1.5 mg/dL; unavailable urinary Na data, inadequate data of tissue Doppler echocardiography, carotid ultrasound, or baPWV; ejection fraction of <55% after echocardiography
Sun, N. (2015)	newly diagnosed hypertensives, untreated hypertensives or patients with a 1 to 5 year history of hypertensives who had stopped taking anti-hypertensive drugs for 1 month	use of anti-hypertensive drugs; secondary hypertension; hypertensive emergency; hypertensive urgency; acute coronary syndrome; severe arrhythmias; DM; stroke; CKD
Han, W. (2017)	hypertensive adults	any secondary cause of hypertension; hypertension emergencies; serious arrhythmia; peripheral arterial disease; heart failure; impaired renal function with plasma creatinine ≥150 μmol/L; rheumatic & autoimmune diseases; malignancies
**2. Observational Studies with Follow up (>1 Time Points)**		
Jung, S. (2019)	adults aged >40	history of heart disease, stroke, and/or cancer; anti-hypertensive drugs; diabetes mellitus; dyslipidemia; implausible dietary intake reported (< 500 or > 4000 kcal/day); missing general characteristic data from the baseline visit
**Common Carotid Arterial Elasticity (Young’s Elastic Modulus, Stiffness Index, Arterial Compliance)**		
Ferreira-Sae, M.C. (2011)	hypertensive adults	age <18 years; neoplastic disease; secondary hypertension
**Arteriosclerosis—Interventional studies**		
**cfPWV**		
Seals, D.R. (2001)	postmenopausal women, ≥50 years, high normal SBP or Stage 1 hypertension	anti-hypertensive drugs; other chronic disease; low-Na diet or regular exercise during the preceding 2 years; smoking
Dickinson, K.M. (2009)	overweight/obese, mild hypertensive adults	metabolic disease; CVD; SBP >160 mm Hg at screening; significant weight loss in the preceding 6 months (>2 kg); BMI < 27 or > 40; use of anti-hypertensive drugs
He, F.J. (2009)	hypertensive adults	anti-hypertensive drugs; secondary cause of hypertension; impaired renal function; previous stroke; ischemic heart disease; heart failure; diabetes mellitus; malignancy; liver disease; pregnancy or lactation or on oral contraceptive pills
Pimenta, E. (2009)	resistant hypertensive adults on stable anti-hypertensive drugs	history of atherosclerotic disease (in the previous 6 months); congestive heart failure; diabetes mellitus on insulin treatment; office blood pressure > 160/100 mm Hg
Todd, A.S. (2010)	Pre-hypertensive or hypertensive, non-obese adults or in anti-hypertensive drugs	age >65; smoking; history of CVD or diabetes mellitus or renal disease
Todd, A.S. (2012)	normotensives, non-obese adults	age >65; antihypertensive medication; smoking; history of cardiovascular disease or diabetes mellitus or renal disease
McMahon, E.J. (2013)	hypertensive adult patients with stage 3 or 4 CKD (GFR 15–59 mL/min per 1.73 m^2^), non-dialyzed, non-transplanted	salt-wasting CKD, pregnant or breastfeeding, current prescription of medications providing 0.20 mmol sodium per day, life expectancy,6 months, current involvement in another intervention study, or insufficient mental or physical capacity to adhere to the study protocol.
Dickinson, K.M. (2014)	overweight or obese subjects	diabetes mellitus; dyslipidemia; inflammatory bowel disease; pulmonary disease or vasculitis
Gijsbers, L. (2015)	untreated prehypertensives, aged 40–80	smoking; diabetes mellitus, CVD; gastrointestinal, liver or renal diseases; BMI > 40; use of drugs known to affect the cardiovascular system; use of nutritional supplements, an energy-restricted or medically prescribed diet; unstable body weight in past 2 months; alcohol use over 21 (women) or 28 (men) consumptions/week; pregnancy or lactation
Suckling, F.J. (2016)	untreated hypertensive adults with diet-controlled type 2 diabetes mellitus or impaired glucose tolerance	any secondary causes of hypertension, impaired renal function (plasma creatinine >150 μmol), uncontrolled heart failure, ischemic heart disease, previous stroke, active malignancy or liver disease, pregnancy, breast feeding, or oral contraceptive drugs
van der Graaf, A.M. (2016)	women with history of preeclampsia or history of healthy former pregnancy	renal disease; diabetes mellitus or a history of gestational diabetes; obesity; use of anti-hypertensive drugs; pregnancy; lactation; postmenopausal status; use of oral contraceptives
Muth, B.J. (2017)	healthy, normotensive adults	history of hypertension; CVD; malignancy; diabetes mellitus; renal impairment; obesity; smoking
**Aortic PWV (Other than cfPWV)**		
Avolio, A.P. (1986)	healthy normotensive adults & children	N/AV
**hfPWV**		
Rhee, M.Y. (2016)	normotensive & hypertensive adults	stage 2 and 3 hypertension; secondary hypertension; angina pectoris; myocardial infarction; congestive cardiac failure; stroke; diabetes mellitus; CKD
**baPWV**		
Wang, Y. (2015)	mild hypertensive adults	stage 2 hypertension; history of clinical CVD; CKD; diabetes mellitus; use of anti-hypertensive drugs; high alcohol intake
**Arterial Elasticity (Arterial Compliance)**		
Creager, M.A. (1991)	normotensive men	hematologic, renal, or hepatic dysfunction
Gates, P.E. (2004)	hypertensive adults (stage 1), older than 50	use of anti-hypertensive drugs; abnormal blood chemistry; positive ECG-monitored exercise test; ankle–brachial index > 0.9; presence of plaque on ultrasound interrogation of the carotid and femoral arteries; smoking for previous 2 years; BMI < 35; consumption of a low-Na diet; not in postmenopausal if female (amenorrheic for at least 2 years)
**Arterial Remodeling—Observational Studies**		
**cIMT**		
**1. Observational Cross-Sectional Studies**		
Ferreira-Sae, M.C. (2011)	hypertensive adults	age <18 years; neoplastic disease; secondary hypertension
Njoroge, J.N. (2011)	overweight or obese, physically inactive adults	diabetes mellitus; anti-hypertensive drugs or average baseline SBP of ≥140 or DBP ≥ 90 mmHg; cholesterol lowering or anti-psychotic or vasoactive drugs; use of vasoactive devices; pregnancy or lactation
García-Ortiz, L. (2012)	primary care patients aged 30–80	cardiovascular and/or cerebrovascular disease
Lee, S.K. (2015)	non-hypertensive individuals, with no use of anti-hypertensive drugs	age >70 years; preexisting CVD including significant valvular heart diseases and arrhythmia; chronic renal disease or a serum creatinine level > 1.5 mg/dL; unavailable urinary Na data, inadequate data of tissue Doppler echocardiography, carotid ultrasound, or baPWV; ejection fraction of <55% after echocardiography
Ustundag, S. (2015)	ambulatory adult patients, in stage 2–4 CKD	BMI < 35 kg/m2; diabetes mellitus; salt-losing nephropathy or history of malignancy or cardio-cerebrovascular disease or any acute disease
Dai, X.W. (2016)	Asian adults, via subject referral and community advertisement	hospital-confirmed diabetes mellitus; CVD; renal failure; CKD; cancer
Mazza, E. (2018)	adults aged ≥65, not suffering from any debilitating diseases	history of CVD or thyroid dysfunction or excessive alcohol consumption; use of dietary supplements & psychotropic drugs
**2. Observational studies with follow up (>1 time points)**		
Jung, S. (2019)	adults aged >40	history of heart disease, stroke, and/or cancer; anti-hypertensive drugs; diabetes mellitus; dyslipidemia; implausible dietary intake reported (< 500 or > 4000 kcal/day); missing general characteristic data from the baseline visit
**Arterial Remodeling—Interventional Studies**		
**Right Branchial Artery & Common Carotid Artery Diameter**		
Benetos, A. (1992)	actively working, mild to moderate hypertensive adults	cardiac, neurologic or renal involvement or arteriosclerosis obliterans of the lower limbs
**Atheromatosis—Observational Studies**		
**Carotid Plaques**		
Dai, X.W. (2016)	Asian adults, via subject referral and community advertisement	hospital-confirmed diabetes mellitus; CVD; renal failure; CKD; cancer
Mazza, E. (2018)	adults aged ≥65, not suffering from any debilitating diseases	history of CVD or thyroid dysfunction or excessive alcohol consumption; use of dietary supplements & psychotropic drugs
Abbreviations: CKD: chronic kidney disease; CVD: cardiovascular disease; BMI: body mass index; Na: Sodium; N/AV: not available.		

## References

[1] Lorenz M.W., Sitzer M., Markus H.S., Bots M.L., Rosvall M. (2007). Prediction of clinical cardiovascular events with carotid intima-media thickness: A systematic review and meta-analysis. Circulation.

[2] Nambi V., Chambless L., He M., Folsom A.R., Mosley T., Boerwinkle E., Ballantyne C.M. (2012). Common carotid artery intima-media thickness is as good as carotid intima-media thickness of all carotid artery segments in improving prediction of coronary heart disease risk in the Atherosclerosis Risk in Communities (ARIC) study. Eur. Heart J..

[3] Vlachopoulos C., Xaplanteris P., Aboyans V., Brodmann M., Cífková R., Cosentino F., De Carlo M., Gallino A., Landmesser U., Laurent S. (2015). The role of vascular biomarkers for primary and secondary prevention. A position paper from the European Society of Cardiology Working Group on peripheral circulation: Endorsed by the Association for Research into Arterial Structure and Physiology (ARTERY) Society. Atherosclerosis.

[4] Powles J., Fahimi S., Micha R., Khatibzadeh S., Shi P., Ezzati M., Engell R.E., Lim S.S., Danaei G., Mozaffarian D. (2013). Global, regional and national sodium intakes in 1990 and 2010, a systematic analysis of 24h urinary sodium excretion and dietary surveys worldwide. BMJ Open.

[5] WHO (2012). Guideline: Sodium Intake for Adults and Children.

[6] Mozaffarian D., Singh G.M., Powles J. (2014). Sodium and cardiovascular disease. N. Engl. J. Med..

[7] Strazzullo P., D’Elia L., Kandala N.B., Cappuccio F.P. (2009). Salt intake, stroke, and cardiovascular disease: Meta-Analysis of prospective studies. BMJ.

[8] Aburto N.J., Ziolkovska A., Hooper L., Elliott P., Cappuccio F.P., Meerpohl J.J. (2013). Effect of lower sodium intake on health: Systematic review and meta-analyses. BMJ.

[9] Mente A., O’Donnell M., Rangarajan S., Dagenais G., Lear S., McQueen M., Diaz R., Avezum A., Lopez-Jaramillo P., Lanas F. (2016). Associations of urinary sodium excretion with cardiovascular events in individuals with and without hypertension: A pooled analysis of data from four studies. Lancet.

[10] Graudal N., Jürgens G., Baslund B., Alderman M.H. (2014). Compared with usual sodium intake, low- and excessive-sodium diets are associated with increased mortality: A meta-analysis. Am. J. Hypertens..

[11] Saulnier P.J., Gand E., Hadjadj S., Surdiagene Study Group (2014). Sodium and cardiovascular disease. N. Engl. J. Med..

[12] O’Donnell M.J., Yusuf S., Mente A., Gao P., Mann J.F., Teo K., McQueen M., Sleight P., Sharma A.M., Dans A. (2011). Urinary sodium and potassium excretion and risk of cardiovascular events. JAMA.

[13] O’Donnell M., Mente A., Rangarajan S., McQueen M.J., Wang X., Liu L., Yan H., Lee S.F., Mony P., Devanath A. (2014). Urinary Sodium and Potassium Excretion, Mortality, and Cardiovascular Events. N. Engl. J. Med..

[14] Alderman M.H., Cohen H.W. (2012). Dietary sodium intake and cardiovascular mortality: Controversy resolved?. Am. J. Hypertens..

[15] Jung S., Kim M.K., Shin J., Choi B.Y., Lee Y.H., Shin D.H., Shin M.H. (2019). High sodium intake and sodium to potassium ratio may be linked to subsequent increase in vascular damage in adults aged 40 years and older: The Korean multi-rural communities cohort (MRCohort). Eur. J. Nutr..

[16] Lee S.K., Kim J.S., Kim S.H., Kim Y.H., Lim H.E., Kim E.J., Park C.G., Cho G.Y., Kim J., Baik I. (2015). Sodium Excretion and Cardiovascular Structure and Function in the Nonhypertensive Population: The Korean Genome and Epidemiology Study. Am. J. Hypertens..

[17] Pimenta E., Gaddam K.K., Oparil S., Aban I., Husain S., Dell’Italia L.J., Calhoun D.A. (2009). Effects of dietary sodium reduction on blood pressure in subjects with resistant hypertension: Results from a randomized trial. Hypertension.

[18] Todd A.S., Macginley R.J., Schollum J.B., Williams S.M., Sutherland W.H., Mann J.I., Walker R.J. (2012). Dietary sodium loading in normotensive healthy volunteers does not increase arterial vascular reactivity or blood pressure. Nephrology.

[19] Dai X.W., Wang C., Xu Y., Guan K., Su Y.X., Chen Y.M. (2016). Urinary Sodium and Potassium Excretion and Carotid Atherosclerosis in Chinese Men and Women. Nutrients.

[20] Mazza E., Ferro Y., Lamprinoudi T., Gazzaruso C., Doldo P., Pujia A., Montalcini T. (2018). Relationship between high sodium and low PUFA intake and carotid atherosclerosis in elderly women. Exp. Gerontol..

[21] Moher D., Liberati A., Tetzlaff J., Altman D.G. (2009). Preferred reporting items for systematic reviews and meta-analyses: The PRISMA statement. BMJ.

[22] Polonia J., Maldonado J., Ramos R., Bertoquini S., Duro M., Almeida C., Ferreira J., Barbosa L., Silva J.A., Martins L. (2006). Estimation of salt intake by urinary sodium excretion in a Portuguese adult population and its relationship to arterial stiffness. Rev. Port. Cardiol..

[23] García-Ortiz L., Recio-Rodríguez J.I., Rodríguez-Sánchez E., Patino-Alonso M.C., Agudo-Conde C., Rodríguez-Martín C., Castaño-Sánchez C., Runkle I., Gómez-Marcos M.A. (2012). Sodium and potassium intake present a J-shaped relationship with arterial stiffness and carotid intima-media thickness. Atherosclerosis.

[24] Kotliar C., Kempny P., Gonzalez S., Castellaro C., Forcada P., Obregon S., Cavanagh E., Chiabaut Svane J., Casarini M.J., Rojas M. (2014). Lack of RAAS inhibition by high-salt intake is associated with arterial stiffness in hypertensive patients. J. Renin Angiotensin Aldosterone Syst..

[25] Polonia J., Monteiro J., Almeida J., Silva J.A., Bertoquini S. (2016). High salt intake is associated with a higher risk of cardiovascular events: A 7.2-year evaluation of a cohort of hypertensive patients. Blood Press Monit..

[26] Strauss M., Smith W., Kruger R., Van der Westhuizen B., Schutte A.E. (2018). Large artery stiffness is associated with salt intake in young healthy black but not white adults: The African-PREDICT study. Eur. J. Nutr..

[27] Triantafyllou A., Anyfanti P., Gkaliagkousi E., Zabulis X., Vamvakis A., Gkolias V., Petidis K., Aslanidis S., Douma S. (2018). Association of Urinary Sodium Excretion with Vascular Damage: A Local Kidney Effect, Rather Than a Marker of Generalized Vascular Impairment. Int. J. Hypertens..

[28] Nerbass F.B., Pecoits-Filho R., McIntyre N.J., Shardlow A., McIntyre C.W., Taal M.W. (2015). Reduction in sodium intake is independently associated with improved blood pressure control in people with chronic kidney disease in primary care. Br. J. Nutr..

[29] Siriopol D., Covic A., Iliescu R., Kanbay M., Tautu O., Radulescu L., Mitu O., Salaru D., Dorobantu M. (2018). Arterial stiffness mediates the effect of salt intake on systolic blood pressure. J. Clin. Hypertens. (Greenwich).

[30] Sonoda H., Takase H., Dohi Y., Kimura G. (2012). Factors associated with brachial-ankle pulse wave velocity in the general population. J. Hum. Hypertens..

[31] Sun N. (2015). Relationship of 24-h urinary sodium excretion with blood pressure, arterial distensibility, and urine albumin in Chinese hypertensive patients. Eur. Heart J. Suppl..

[32] Han W., Han X., Sun N., Chen Y., Jiang S., Li M. (2017). Relationships between urinary electrolytes excretion and central hemodynamics, and arterial stiffness in hypertensive patients. Hypertens. Res..

[33] Ferreira-Sae M.C., Cipolli J.A., Cornélio M.E., Matos-Souza J.R., Fernandes M.N., Schreiber R., Costa F.O., Franchini K.G., Rodrigues R.C., Gallani M.C. (2011). Sodium intake is associated with carotid artery structure alterations and plasma matrix metalloproteinase-9 upregulation in hypertensive adults. J. Nutr..

[34] Seals D.R., Tanaka H., Clevenger C.M., Monahan K.D., Reiling M.J., Hiatt W.R., Davy K.P., DeSouza C.A. (2001). Blood pressure reductions with exercise and sodium restriction in postmenopausal women with elevated systolic pressure: Role of arterial stiffness. J. Am. Coll. Cardiol..

[35] Dickinson K.M., Keogh J.B., Clifton P.M. (2009). Effects of a low-salt diet on flow-mediated dilatation in humans. Am. J. Clin. Nutr..

[36] He F.J., Marciniak M., Visagie E., Markandu N.D., Anand V., Dalton R.N., MacGregor G.A. (2009). Effect of modest salt reduction on blood pressure, urinary albumin, and pulse wave velocity in white, black, and Asian mild hypertensives. Hypertension.

[37] Todd A.S., MacGinley R.J., Schollum J.B., Johnson R.J., Williams S.M., Sutherland W.H., Mann J.I., Walker R.J. (2010). Dietary salt loading impairs arterial vascular reactivity. Am. J. Clin. Nutr..

[38] McMahon E.J., Bauer J.D., Hawley C.M., Isbel N.M., Stowasser M., Johnson D.W., Campbell K.L. (2013). A randomized trial of dietary sodium restriction in CKD. J. Am. Soc. Nephrol..

[39] Dickinson K.M., Clifton P.M., Keogh J.B. (2014). A reduction of 3 g/day from a usual 9 g/day salt diet improves endothelial function and decreases endothelin-1 in a randomised cross_over study in normotensive overweight and obese subjects. Atherosclerosis.

[40] Gijsbers L., Dower J.I., Mensink M., Siebelink E., Bakker S.J., Geleijnse J.M. (2015). Effects of sodium and potassium supplementation on blood pressure and arterial stiffness: A fully controlled dietary intervention study. J. Hum. Hypertens..

[41] Suckling R.J., He F.J., Markandu N.D., MacGregor G.A. (2016). Modest Salt Reduction Lowers Blood Pressure and Albumin Excretion in Impaired Glucose Tolerance and Type 2 Diabetes Mellitus: A Randomized Double-Blind Trial. Hypertension.

[42] van der Graaf A.M., Paauw N.D., Toering T.J., Feelisch M., Faas M.M., Sutton T.R., Minnion M., Lefrandt J.D., Scherjon S.A., Franx A. (2016). Impaired sodium-dependent adaptation of arterial stiffness in formerly preeclamptic women: The RETAP-vascular study. Am. J. Physiol. Heart Circ. Physiol..

[43] Muth B.J., Brian M.S., Chirinos J.A., Lennon S.L., Farquhar W.B., Edwards D.G. (2017). Central systolic blood pressure and aortic stiffness response to dietary sodium in young and middle-aged adults. J. Am. Soc. Hypertens..

[44] Avolio A.P., Clyde K.M., Beard T.C., Cooke H.M., Ho K.K., O’Rourke M.F. (1986). Improved arterial distensibility in normotensive subjects on a low salt diet. Arteriosclerosis.

[45] Rhee M.Y., Kim J.H., Na S.H., Chung J.W., Bae J.H., Nah D.Y., Gu N., Kim H.Y. (2016). Elevation of heart-femoral pulse wave velocity by short-term low sodium diet followed by high sodium diet in hypertensive patients with sodium sensitivity. Nutr. Res. Pract..

[46] Wang Y., Mu J.J., Geng L.K., Wang D., Ren K.Y., Guo T.S., Chu C., Xie B.Q., Liu F.Q., Yuan Z.Y. (2015). Effect of salt intake and potassium supplementation on brachial-ankle pulse wave velocity in Chinese subjects: An interventional study. Braz. J. Med. Biol. Res..

[47] Creager M.A., Roddy M.A., Holland K.M., Hirsch A.T., Dzau V.J. (1991). Sodium depresses arterial baroreceptor reflex function in normotensive humans. Hypertension.

[48] Gates P.E., Tanaka H., Hiatt W.R., Seals D.R. (2004). Dietary sodium restriction rapidly improves large elastic artery compliance in older adults with systolic hypertension. Hypertension.

[49] Njoroge J.N., Khoudary S.R., Fried L.F., Barinas-Mitchell E., Sutton-Tyrrell K. (2011). High urinary sodium is associated with increased carotid intima-media thickness in normotensive overweight and obese adults. Am. J. Hypertens..

[50] Ustundag S., Yilmaz G., Sevinc C., Akpinar S., Temizoz O., Sut N., Ustundag A. (2015). Carotid intima media thickness is independently associated with urinary sodium excretion in patients with chronic kidney disease. Ren. Fail..

[51] Benetos A., Xiao Y.Y., Cuche J.L., Hannaert P., Safar M. (1992). Arterial effects of salt restriction in hypertensive patients. A 9-week, randomized, double-blind, crossover study. J. Hypertens..

[52] Felder R.A., White M.J., Williams S.M., Jose P.A. (2013). Diagnostic tools for hypertension and salt sensitivity testing. Curr. Opin. Nephrol. Hypertens..

[53] D’Elia L., Galletti F., La Fata E., Sabino P., Strazzullo P. (2018). Effect of dietary sodium restriction on arterial stiffness: Systematic review and meta-analysis of the randomized controlled trials. J. Hypertens..

[54] Edwards D.G., Farquhar W.B. (2015). Vascular effects of dietary salt. Curr. Opin. Nephrol. Hypertens..

[55] Simon G. (2003). Experimental evidence for blood pressure-independent vascular effects of high sodium diet. Am. J. Hypertens..

[56] Elijovich F., Weinberger M.H., Anderson C.A., Appel L.J., Bursztyn M., Cook N.R., Dart R.A., Newton-Cheh C.H., Sacks F.M., Laffer C.L. (2016). Salt Sensitivity of Blood Pressure: A Scientific Statement from the American Heart Association. Hypertension.

